# Measurement, spatial differences and driving effects of well-being levels in China

**DOI:** 10.1371/journal.pone.0311291

**Published:** 2024-10-01

**Authors:** Yumeng Zhang, Chongyang Zhong, Yin Wang

**Affiliations:** 1 School of Urban and Regional Science, Shanghai University of Finance and Economics, Shanghai, China; 2 School of Economics, Nanjing University of Finance and Economics, Jiangsu, Nanjing, China; 3 College of Urban and Environmental Sciences, Northwest University, Shaanxi, Xi’an, China; Huazhong University of Science and Technology, CHINA

## Abstract

As the largest developing country, China has accumulated enormous material wealth since its reform and opening-up policy. How to effectively evaluate the level of well-being in China has become a meaningful research endeavor. Using the entropy method, Dagum Gini coefficient and Logarithmic Mean Divisia Index (LMDI) decomposition methods, the study examines the spatial and temporal distribution characteristics, spatial differences and driving effects of provincial well-being levels from 2007 to 2020. The results of this study suggest that the level of well-being as a whole, as well as in the eastern, central and western regions increased significantly over the period, with an “east-to-west decreasing” distribution in China. In terms of the pattern of inter-provincial distribution, although the level of well-being in the central and western regions has improved at a faster rate, most provinces in the eastern region have always been among the leading teams on the path of livelihood development. There is still enormous room for improvement in the level of well-being in the central and western provinces. The overall differences in the development of well-being in China, as well as intra-regional and inter-regional differences among the three major regions, showed a narrowing trend. Intra-regional differences in the development of well-being are greatest in the western region, and inter-regional differences in the development of well-being are greatest in the eastern and western regions. Inter-regional differences are the main reason for the spatial differences in well-being among China’s provinces. The combination of economic, social, ecological and technological effects has led to a gradual increase in the level of well-being over the sample period. Among them, economic, social and technological effects have a clear positive driving effect on the increase of well-being levels, while ecological effect have a certain negative driving influence.

## 1 Introduction

Governments and research institutions have prioritized developing and refining the Sustainable Development Goals and improving human well-being over the past decades. Since the reform and opening-up, China has accumulated huge material wealth, remarkably increased people’s income and consumption levels, and significantly improved the quality of people’s lives. In October 2020, the Chinese government put forward the goal of “making more obvious and substantial progress in common prosperity for all the people by 2035,” expressing its earnest expectation and policy direction for improving people’s well-being. However, despite its achievements, China still faces huge challenges in narrowing the income gap between residents and achieving universal access to basic public services [1], which is not conducive to improving residents’ well-being and happiness [2]. The real causes of these problems can be considered from two aspects. First, China is a vast country and there are inherent differences between regions in terms of historical evolution, resource endowment and environmental carrying capacity. The locational conditions for economic and social development in each region are markedly different, and after a long-term cyclical and cumulative process, there has been a significant development gap among different regions. Second, China’s socialist market economy system is still not mature enough. There is still huge room for improvement in the allocation of regional resources, optimization of industrial structure and enterprise system and integrated development of urban-rural areas. Against this background, based on the reality of unbalanced development in various regions of China, this paper constructs an index system and measures and analyzes the level of well-being, spatial differences and driving effects. This is of great significance for grasping the development of residents’ livelihood in China and improving the level of well-being in various regions.

Human well-being is a concept that contains multiple dimensions. The human development index (HDI), proposed by the United Nations in 1990, is currently the most widely known index of well-being. HDI is calculated from three indicators: life expectancy, adult literacy and GDP per capita, corresponding to a person’s longevity, knowledge and standard of living [3]. However, because of the narrow range of measurement, simple calculation method and deviation of the results, this index is considered inadequate [4, 5]. Fortunately, index systems of human well-being that include both subjective and objective elements have been proposed in subsequent related studies. Combining the concept of well-being economy, Fioramonti et al. [6] proposes that we should not only focus on the increase of material wealth (e.g., GDP), but also consider the importance of economic and social factors in realizing well-being. It is necessary to build a well-being system that covers social equity, physical and mental health, public education, ecological environment and urban-rural balance. Meanwhile, some empirical conclusions on the influencing factors of human well-being can also provide the references for the construction of well-being index system. For example, the welfare policy framework of the Organization for Economic Co-operation and Development (OECD) proposes some key determinants of well-being, including income and wealth, employment, housing, knowledge and skills, security and work-life balance [7]. The contribution of external indexes, such as natural ecosystems and social environments, to well-being has also been recognized by academic research [8]. In addition, scholars who study issues related to well-being also point out that the factors affecting residents’ well-being are multi-dimensional, including not only revenue [9–11], but also non-income factors, such as public services, social environment and ecological environment [12]. In conclusion, the discussion of the connotation and influencing factors of well-being in these studies above provides some references for us to construct the index system of China’s well-being level. Specifically, in order to provide a more realistic picture of well-being level, the scope and nature of the index should be considered together. The scope of the index should not only consider material wealth and income, but also incorporate elements of social well-being (such as better public services, greater social stability and lower inequality) that can enhance the sense of well-being and actual fulfillment of the population. Of course, we do not deny that more abundant material production can improve the living standards of residents. But some studies have shown little correlation between growth and well-being after certain thresholds of basic needs are met [13–15]. Moreover, the nature of the index considers an increase in aggregation in the sense of scale and a reduction in inequality in the sense of sharing. It should be noted that it is difficult to determine a uniform and universally applicable measurement standard in the design of well-being index system [16]. Therefore, we believe that constructing a system of China’s well-being index, it is necessary to draw on the experiences and practices of existing studies, as well as to consider China’s economic and social development and availability of data for the relevant indexes.

From the existing studies, the commonly used methods for calculating the well-being index can be categorized into subjective and objective empowerment methods. Commonly used subjective empowerment methods include the analytic hierarchy process and the expert-scoring method. Kibria et al. [17] calculated five categories of indices in human well-being using analytic hierarchy process and summed them to obtain a composite well-being index. There are also studies that have used questionnaires to obtain well-being scores [18, 19]. Since people’s perceptions of the “good life” can vary widely [20], the determination of weights can be biased by subjective judgments. It has been noted that the calculation methodology used for the HDI is also highly subjective [21, 22]. Different from the subjective weighting methods, objective weighting methods can determine the weights based on the relationship between data and distribution characteristics of the data. Its judgment results do not rely on people’s subjective judgment and have a strong mathematical and scientific basis [23]. Commonly used methods include principal component analysis and entropy method. Among them, principal component analysis allows the data to be downscaled so that the initial set of indicators is reduced to a linear combination of an equal number of uncorrelated indicators [24, 25]. However, this method also suffers from limitations such as over-reliance on correlation for weight assignment [26, 27] and easy missing of important information [28]. Compared with it, the entropy method can calculate the weight of indicators according to the size of information carried by the data, and better provide the basis for the comprehensive evaluation of multiple indicators [29, 30]. In recent years, several researchers have used the methodology to conduct studies around issues such as poverty [31] and ecology [29, 32], which are closely related to the theme of well-being.

In general, the connotation of well-being and the index system is gradually expanding, which provides useful reference for this paper to measure and evaluate the level of well-being in China. There is still much room for improvement in existing research. In this regard, this study is expected to make academic contributions in the following three areas.

First, many studies have explored the connotation and realization path of well-being or happiness of Chinese residents. A little literature attempts to measure and analyze the well-being level, which still suffers from a short sample period and unreasonable index weights. As well as the lack of consideration of the actual problems of China’s economic and social development in the design of index, the conclusions lack representativeness to a certain extent. This paper considers the empirical practices of existing studies and the policy practices of the Chinese Government in its efforts to narrow the internal development gap and raise the level of common prosperity. Based on a system of indicators for evaluating the development of well-being in China in terms of scale and sharing, the study then uses the entropy method to measure the level of well-being in China from 2007 to 2020. It also comprehensively evaluates the state of well-being in China from the perspectives of the nation, the inter-provincial pattern and the three major regions, respectively. All the above work has at least two aspects of academic contribution. On the one hand, the 16 economic and social indicators selected in this paper cover the five major areas of livelihood and spiritual affluence, access to public services, environmental stability, group sharing, and urban-rural sharing. It can provide a new reference for the development of the evaluation system of livelihood and well-being in developing countries. On the other hand, through quantitative evaluation from multiple perspectives, the study can provide empirical evidence for grasping the spatial and temporal distribution characteristics of China’s well-being development.

Second, the existing quantitative analysis of China’s well-being level is mainly based on the descriptive statistics of specific survey data (such as data from *China General Social Survey* and *China Family Panel Studies*). There is a paucity of studies examining the spatial differences in the level and sources of well-being based on continuous panel data, which is difficult to characterize the spatial imbalances of China’s well-being level and its evolutionary trend. There are obvious development differences between China’s regions due to natural conditions, resource endowments, cultural traditions and other factors (in fact, this is a common problem in developing countries). Therefore, when evaluating the development level of well-being, spatial differences should be the focus of investigation, and this paper adopts the Dagum Gini coefficient and its decomposition method to solve this problem. This method can not only reveal the overall differences and annual variation characteristics, but also decompose the overall differences into intra-regional differences, inter-regional differences and super-variable density. It also identifies the main sources of spatial differences in the level of well-being by calculating the contribution rates of the three. Through the above analysis, the study can not only provide scientific suggestions for promoting the coordinated improvement of well-being levels, but also supplement the evaluation study of regional development differences.

Finally, existing studies give some discussion of the causal relationship between well-being level and other economic and social factors. They mainly analyze it through survey data of individual years. The literature examining the driving effects behind the dynamic evolution of well-being levels is still relatively scarce, and it is not possible to identify the driving effects that cause changes in well-being levels from the perspective of structural decomposition. The study uses Kaya identity to deconstruct China’s well-being index from four dimensions: economic effect, social effect, ecological effect and technological effect. It also utilizes the LMDI decomposition method to identify the driving effects of dynamic changes in well-being level. The method has the advantages of zero residuals, strong interpretability and applicability to many types of variable decompositions, and is now widely used in energy, environment, and other research fields [33–35]. Unlike the previous studies, this paper utilizes the LMDI decomposition method to examine the driving effects of well-being at the provincial level in China. Obviously, this is a useful attempt to use the method for the study of other economic and social issues, and can provide methodological references for identifying the internal drivers of economic and social development in a country or a region. It provides direct and clear empirical evidence for exploring the direction and path of China’s sustainable improvement of well-being levels.

The rest of the paper is organized as follows. Section 2 discusses the methodology, sample and data sources. Section 3 provides a comprehensive evaluation of well-being level. The spatial differences and driving effects of well-being are presented in Sections 4 and 5, respectively, followed by the conclusion and discussion in Section 6 and 7.

## 2 Research design

### 2.1 Methods

#### 2.1.1 Measurements of the well-being index

Common weighting methods can be divided into subjective and objective weighting methods. Among them, the subjective weighting method is highly dependent on the experience of experts, whereas the weighting process of objective weighting methods, such as principal component analysis, is easily disturbed by outliers, which may lead to individual weights deviating from the normal range. In view of this, this paper chooses the entropy evaluation method, which can overcome the above problems to measure well-being index (WI) of China. This method can fully consider the numerical information of the index itself and better resolve the internal conflict between embedding criteria in multi-attribute decision-making problems so that the evaluation results are more accurate [30]. In this paper, the larger the entropy value of an index, the smaller the degree of variation of its observed value; the less information provided in the measurement of WI, the smaller the weight. On the contrary, the smaller the entropy value, the stronger the importance and weight of the WI measurement. The specific calculation process is as follows.

First, we calculate the proportion (*B*_*ij*_) of province *i* under index *j* in the index system, where *n* indicates the number of provinces.

$$B_{ij}=x_{ij}/\sum_{i=1}^{n}x_{ij}$$ (1)

Second, we calculate the information entropy (*g*_*j*_) of the index *j*.


$$g_{j}=-(1/\ln\;n)\sum_{i=1}^{n}B_{ij}\;\ln\;B_{ij}$$ (2)

Third, we calculate the difference coefficient (*e*_*j*_) of the index *j*.


$$e_{j}=1-g_{j}$$ (3)

Next, we calculate the weight (*a*_*j*_) of the index *j*, where *m* indicates the number of indexes.


$$a_{j}=e_{j}/\sum_{j=1}^{m}e_{j}$$ (4)

Finally, WI can be calculated using the dimensionless index *Z*_*j*_ and its weight *a*_*j*_ according to Eq (5). Based on the calculation results of each index, well-being scale index and well-being sharing index are synthesized. It should be noted that since this paper does not use the entropy method to calculate each of the three indices independently, the well-being scale index and the well-being sharing index are used as components that together make up the well-being index.


$$WI_{j}=\sum_{j=1}^{n}Z_{j}a_{j}/\sum_{j=1}^{n}a_{j}\times 100$$ (5)

The calculation method of *Z* is divided into two steps. First, according to the criterion of whether the larger the better or the smaller the better for the evaluation of well-being, the index is divided into positive index or reverse index. Second, calculate the dimensionless index value (*Z*) of forward index and reverse index according to Eq (6).


$$Forward\;index:\;Z_{j}=\frac{X_{j}-X_{\min}^{j}}{X_{\max}^{j}-X_{\min}^{j}}\;Reverse\;index:\;Z_{j}=\frac{X_{\max}^{j}-X_{j}}{X_{\max}^{j}-X_{\min}^{j}}$$ (6)

#### 2.1.2 Measurement of spatial differences of well-being levels

In international statistical work, the traditional Gini index method, proposed by the Italian economist Gini in the early twentieth century based on the Lorenz curve, is often used to characterize the degree of income inequality in a country or region. However, this method has obvious limitations. On the one hand, the traditional Gini index is usually calculated based on the area enclosed by the Lorenz curve and the absolute average income curve, but the corresponding Gini indexes may be the same for different Lorenz curves. On the other hand, since the Gini index can only reflect the size of the overall difference, it is difficult to truly reflect the differences between different regions in the study of countries with obvious regional disparities in economic development. In later studies, some scholars have refined the method or proposed new methods for calculating income inequality [36, 37]. Among them, the Gini index measure proposed by Dagum [36] can both examine the size of the overall differences and decompose the overall differences into intra-area differences, inter-area differences and super-variable density. It not only overcomes the shortcomings of the Thiel index and the traditional Gini index, but also is able to better reveal the sources of spatial differences. Therefore, this paper uses the Dagum Gini index and its decomposition to measure the spatial differences in the level of provincial well-being in China.

Eq (7) shows the calculation method of the overall Gini index. Eq (8) ranks the regions according to the mean of well-being level. *G*_*jj*_ in Eq (9) is the Gini index of region *j*, *G*_*w*_ in Eq (10) is the contribution of regional differences. *G*_*jh*_ in Eq (11), *G*_*nb*_ in Eq (12) and *G*_*t*_ in Eq (13) are the Gini index between *j* and *h* regions, the contribution of net value differences between regions and super-variable density, respectively. Among them, the overall Gini index *G* is equal to the sum of *G*_*w*_, *G*_*nb*_ and *G*_*t*_.


$$G=\frac{\sum_{j=1}^{k}\sum_{h=1}^{k}\sum_{i=1}^{n_{j}}\sum_{r=1}^{n_{h}}\left|y_{ji}-y_{hr}\right|}{2n^{2}\bar{y}}$$ (7)

$${\bar{y}}_{h}\leq \cdots \leq {\bar{y}}_{j}\leq \cdots \leq {\bar{y}}_{k}$$ (8)

$$G_{jj}=\frac{\frac{1}{2{\bar{y}}_{j}}\sum_{i=1}^{n_{j}}\sum_{r=1}^{n_{j}}\left|y_{ji}-y_{jr}\right|}{n_{j}^{2}}$$ (9)

$$G_{w}=\sum_{j=1}^{k}G_{jj}p_{j}s_{j}$$ (10)

$$G_{jh}=\frac{\sum_{i=1}^{n_{j}}\sum_{r=1}^{n_{h}}\left|y_{ji}-y_{hr}\right|}{n_{j}n_{h}({\bar{y}}_{j}+{\bar{y}}_{h}|}$$ (11)

$$G_{nb}=\sum_{j=2}^{k}\sum_{h=1}^{j-1}G_{jh}(p_{j}s_{h}+p_{h}s_{j})D_{jh}$$ (12)

$$G_{t}=\sum_{j=2}^{k}\sum_{h=1}^{j-1}G_{jh}(p_{j}s_{h}+p_{h}s_{j})\left(1-D_{jh}\right)$$ (13)

$$s.t.\;p_{j}=\frac{n_{j}}{\bar{y}},s_{j}=\frac{n_{j}{\bar{y}}_{j}}{n\bar{y}}\left(j=1,2,\cdots ,k\right)$$

*D*_*jh*_ in Eq (14) is the relative impact of WI between the *j* and *h* regions. Eq (15) indicates that by calculating the mathematical expectation of the sum of all sample values with y_*ji*_−*y*_*hr*_>0, the difference *d*_*jh*_ of well-being between *j* and *h* regions can be obtained. Eq (16) is the super-variable first moment *p*_*jh*_, which is obtained by calculating the mathematical expectation of the sum of the sample values of all y_*hr*_−*y*_*ji*_>0 in region *j* and region *h*. *y*_*ji*_ (*y*_*hr*_) is the WI of province *i* (*r*) in region *j* (*h*) and $\bar{y}$ on behalf of its average. *n* and *k* are the number of provinces and regions, *n*_*j*_ and *n*_*h*_ represent the number of provinces in regions *j* and *h*. ${\bar{y}}_{j}$and ${\bar{y}}_{h}$represent the mean value of WI in regions *j* and *h*. *F*_*j*_ (*F*_*h*_) is a function of the cumulative density distribution of the *j* (*h*) region.


$$D_{jh}=\frac{d_{jh}-p_{jh}}{d_{jh}+p_{jh}}$$ (14)

$$d_{jh}=\int_{0}^{\infty }dF_{j}(y)\int_{0}^{y}\left(y-x\right)dF_{h}(x)$$ (15)

$$p_{jh}=\int_{0}^{\infty }dF_{h}(y)\int_{0}^{y}\left(y-x\right)dF_{j}(x)$$ (16)

#### 2.1.3 Driving effects identification of well-being

*(1) Kaya identity*. Proposed by Japanese scholar Yoichi Kaya at the IPCC seminar [38], Kaya identity can decompose CO_2_ emissions into related factors, such as economic growth, energy consumption and population; it has been widely used in resource and environmental fields, such as carbon emissions, energy structure and energy consumption [39].

In view of the convenience of this method for the decomposition of specific factors, this paper introduces Kaya identity into the driving effect decomposition study of dynamic changes in WI and extends it as follows:

$$WI=GDP\times \frac{WI}{GDP}\times \frac{PE}{GDP}\times \frac{GDP}{PE}=G\times S\times E\times T$$ (17)

According to Eq (17), WI can be decomposed into the economic index (*G*), social index (*S*), ecological index (*E*) and technological index (*T*). Among these, economic index (*G*) is measured by deflated real GDP per capita, which depicts the fundamental role of economic growth in improving well-being level. Social index (*S*) is measured by the ratio of well-being level to real GDP per capita, that is, the contribution of unit economic growth to well-being level, which is regarded as the result of social development. Ecological index (*E*) is measured by the ratio of SO_2_ emissions to real GDP per capita, that is, the amount of air pollution per unit of economic growth, which reflects the impact of changes in the ecological environment on well-being level. Technological index (*T*) is measured by the ratio of real GDP per capita to SO_2_ emissions, that is, the amount of economic output an economic entity gains by sacrificing unit environmental quality, which reflects production technology update and resource utilization efficiency and is helpful to analyze the impact of technological progress on well-being level.

*(2) LMDI decomposition method*. LMDI is characterized by path independence, self-adaptability and aggregation consistency. It does not produce residual errors in the decomposition process and has a good ability to deal with zero values [40, 41]. LMDI has both additive and multiplicative forms. To make the decomposition results easy to explain, this paper adopts the sum form.

Let Δ*WI* be the total effect of changes in WI in the base period and year *t*, which is composed of the change effect of economic effect (*Ge*), social effect (*Se*), ecological effect (*Ee*) and technology effect (*Te*). Then, the following LMDI decomposition model can be constructed:

$$\Delta WI=WI_{t}-WI_{0}=G_{e}+S_{e}+E_{e}+T_{e}$$ (18)

$$G_{e}=\sum \frac{\left(WI_{0}^{t}-WI_{i}^{0}\right)}{\left(\ln\;WI_{i}^{t}-\ln\;WI_{i}^{0}\right)}•\ln(\frac{G_{i}^{t}}{G_{i}^{0}})$$ (19)

$$S_{e}=\sum \frac{\left(WI_{0}^{t}-WI_{i}^{0}\right)}{\left(\ln\;WI_{i}^{t}-\ln\;WI_{i}^{0}\right)}•\ln(\frac{S_{i}^{t}}{S_{i}^{0}})$$ (20)

$$E_{e}=\sum \frac{\left(WI_{0}^{t}-WI_{i}^{0}\right)}{\left(\ln\;WI_{i}^{t}-\ln\;WI_{i}^{0}\right)}•\ln(\frac{E_{i}^{t}}{E_{i}^{0}})$$ (21)

$$T_{e}=\sum \frac{\left(WI_{0}^{t}-WI_{i}^{0}\right)}{\left(\ln\;WI_{i}^{t}-\ln\;WI_{i}^{0}\right)}•\ln(\frac{T_{i}^{t}}{T_{i}^{0}})$$ (22)

The decomposition formula of the total effect of changes in WI is shown in Eq (18), and the calculation methods of the four sub-effects are shown in Eqs (19)–(22). A positive total effect indicates a positive change in well-being level and a negative change on the contrary. Positive sub-effects indicate that the change in this aspect has a positive driving effect on WI and vice versa.

### 2.2 Study sample and data sources

This paper takes 31 provincial-level administrative units in China as samples and based on the comparability and availability of index data, the sample period is set as 2007–2020. In terms of data sources, the average salary of internal management and the average salary of employees are obtained from the China Stock Market & Accounting Research Database (CSMAR) and manually sorted according to the place where the enterprise is registered. The other variables are collected from the *China Statistical Yearbook*. For missing values, this paper uses the linear interpolation method or refers to the *Statistical Bulletin on National Economic and Social Development* to supplement.

## 3 Evaluation of well-being levels

### 3.1 Constructing the index system of well-being levels

In March 2021, the Chinese government issued *the 14th Five-Year Plan*, which takes improving well-being level and promoting well-rounded human development as an era task and clearly proposes to “narrow the development gap, let the fruits of development benefit all people broadly and fairly, and constantly enhance people’s sense of gain, happiness and security”. This indicates that improving the level of well-being not only needs increasing in the scale of well-being level but also promoting the degree of sharing in it. Indeed, some studies on measures of well-being levels also follow this line of thought. For example, Fleurbaey and Gaulier [42] constructed a comprehensive social well-being index based on income, including life expectancy, unemployment risk, inequality, labor force and other factors. The ranking results of the national welfare level calculated by this index were obviously different from those obtained by GDP. Jones and Klenow [43] also used similar indexes to measure well-being levels in several countries and the results showed that for most developing economies, inequality would lead to remarkably lower well-being than income. Development and sharing are both key elements of residents’ well-being. The former corresponds to wealth accumulation and monetary aggregate growth, whereas the latter emphasizes equality and equity. Therefore, building index to assess well-being level, we should consider both aggregate abundance and the sharing of progress results. This study refers to both relevant studies [2, 44–46] on residents’ well-being and social welfare and the idea of constructing the HDI and combines the policy direction of the Chinese government’s efforts to narrow the internal development gap and raise the level of common prosperity. In our study, China’s well-being index system includes scale and sharing, specifically covering five aspects of material enjoyment and cultural enrichment, access to public services, environmental stability, community sharing and urban-rural sharing (as shown in Table 1). It should be noted that this paper measures the level of well-being rather than subjective happiness. Although some studies have argued that high income can bring a sense of well-being, we believe that it is inappropriate to consider only income and ignore consumption. Therefore, our index system comprehensively considers external environmental factors such as residents’ income and consumption, public services and social stability. It can more fully and truly reflect the development of well-being.

**Table 1:** Index system for well-being level.

Grade Ⅰ	Grade Ⅱ	Grade Ⅲ	Grade Ⅳ
Well-being level	Scale	Material enjoyment and cultural enrichment	Household income real per capita (CNY)
			Expenditure on culture, education and entertainment real per capita (CNY)
		Access to public services	Urban road area per capita (m^2^)
			Illiteracy rate (%)
			Junior high school student-teacher ratio (teacher = 1)
			Park green area per capita (m^2^)
			Number of beds in medical and health institutions per capita (piece)
		Environmental stability	Inverse of industrial SO_2_ emissions
			Employment injury insurance coverage rate (%)
			Number of urban road lights per capita (pieces)
			Unemployment rate (%)
	Sharing	Community sharing	Average remuneration of management within the enterprise / average remuneration of employees (employees = 1)
			Number of persons working in road transport and postal services / road mileage (persons / km)
			Difference between per capita financial expenditure and the national average for the year
		Urban-rural sharing	Ratio of mobile telephones and computers per 100 households in urban and rural areas (rural = 1)
			Comparison of real consumption levels of urban and rural residents (rural = 1)

On the one hand, in the scale of well-being dimension, real per capita household income and real per capita expenditure on culture, education and entertainment correspond to the material enjoyment and cultural enrichment, respectively. In this paper, they are deflated using the consumer price index. The degree of public service enjoyment is mainly considered from the perspective of education, medical care and environment. Environmental stability includes both ecological and social indexes, such as the inverse of industrial SO_2_ emissions, employment injury insurance coverage rate, the number of urban road lights per capita and unemployment rate. From the nature of specific index, the increase of the junior high school student-teacher ratio, the illiteracy rate and the unemployment rate correspond to the shortage of educational resources, the decrease of the overall human capital level and the decrease of the average income level, which are not conducive to improving the total well-being. On the contrary, the increase in other indexes can improve residents’ sense of gain and happiness to expand the scale of well-being.

On the other hand, in the well-being sharing dimension, community sharing considers the group income gap, the level of coverage of logistics and transportation services and the per capita financial expenditure gap. Among them, the group income gap is expressed by the ratio of the average remuneration of management and employees within an enterprise, reflecting the degree of income inequality among different groups in Chinese society. Although, the ratio of wages between management and workers may reflect the industrialization of a region, or the gap in wage status between the two may not be significant. However, in China’s current economic and social development, the continued widening of the pay gap between corporate executives and ordinary employees has become a concrete manifestation of the widening gap in income distribution [47, 48]. The coverage level of logistics and transportation services is measured by the density of people working in road transportation and postal services, reflecting the convenience of residents in accessing external products and services. The gap in per capita fiscal expenditure is measured by the difference between per capita fiscal expenditure and the national average for the year, reflecting the level of government expenditure in guaranteeing residents’ equitable access to public services. In China, local public services are almost exclusively provided by local governments in the form of public expenditure [49]. Therefore, with relatively limited access to indexes, we have attempted to calculate the difference between per capita fiscal expenditure in each province and the national average for that year, based on local fiscal expenditure data, and used it as an index to measure public service sharing. This has the advantage of being able to capture the important element of local public services in both the scale dimension and the shared dimension, while avoiding the duplication of many indicators. Urban-rural sharing is considered mainly in terms of the gap between the consumption capacity of urban and rural residents and the gap in the level of information technology benefits. It includes two indicators, namely, the contrast between the real consumption levels of urban and rural residents (this paper uses the consumer price index for deflation) and the ratio of mobile telephones and computers per 100 households in urban and rural areas. By the nature of the index, a higher density of people working in road transportation and postal services indicates higher accessibility of logistics and transportation services. The more conducive it is to smooth intermediate paths for residents (especially rural residents) to access all types of goods and affordable services, thus increasing the level of shared well-being. Conversely, an increase in the remaining four indexes will lead to a widening of the gap between group incomes, the gap between levels of per capita financial expenditure as well as the gap between urban and rural residents in terms of their access to consumer and information-based services, thus hindering the sharing of well-being.

### 3.2 Analysis of China’s provincial well-being level

Based on the above index system, this paper utilizes the entropy method to measure the level of inter-provincial well-being in China, and comprehensively evaluates the development of well-being from the overall evolution trends, the provincial distribution patterns and the three major regional perspectives, respectively. The sample provinces were divided into the eastern, central and western regions according to the National Bureau of Statistics of China’s three zone division standards. Among them, the eastern region includes Beijing, Tianjin, Hebei, Liaoning, Shanghai, Jiangsu, Zhejiang, Fujian, Shandong, Guangdong and Hainan. The central region includes Shanxi, Jilin, Heilongjiang, Anhui, Jiangxi, Henan, Hubei and Hunan. The western region includes Inner Mongolia Autonomous Region, Guangxi, Chongqing, Sichuan, Guizhou, Yunnan, Tibet Autonomous Region, Shaanxi, Gansu, Qinghai, Ningxia Hui Autonomous Region and Xinjiang Uygur Autonomous Region. The specific analysis is as follows.

**Fig 1:**
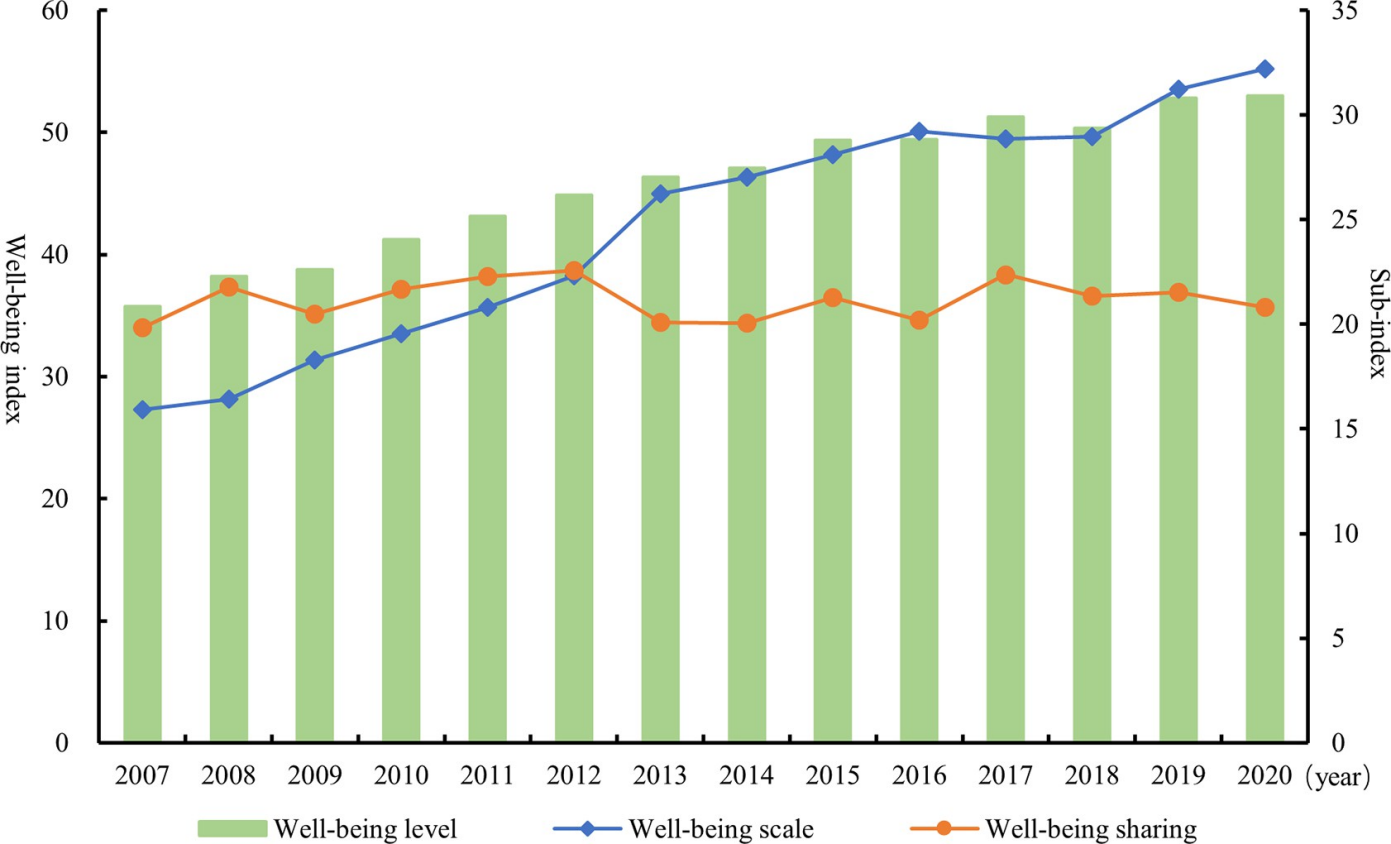
Evolution trend of the mean value of China’s well-being index and its sub-index from 2007 to 2020.

First, in order to grasp the overall characteristics of the evolution of China’s well-being level, this paper plots the evolution trend of the mean value of the well-being index and its sub-indexes from 2007 to 2020 (as shown in Fig 1). The well-being level showed a significant improvement trend during the sample study period, with an average annual growth rate of 3.08%, indicating that the development momentum of the well-being level was generally good. In terms of the sub-indices, the evolution of well-being scale is largely in line with the level of well-being, with an average annual growth rate of 5.58%. In contrast, the growth rate of well-being sharing is lower at 0.37%. Comparing the year-by-year changes of the three indices, the well-being level and well-being scale both increased year by year from 2007 to 2016 and then turned into a fluctuating and increasing trend. Compared with them, the volatility characteristic of well-being sharing is more obvious, and the growth rate is significantly lower than that of well-being level and well-being scale. This means that while increasing the total amount of well-being, accelerating the optimization of the distribution system and promoting the sharing of the fruits of development have become essential for the Chinese Government to sustainably raise the level of well-being.

**Fig 2:**
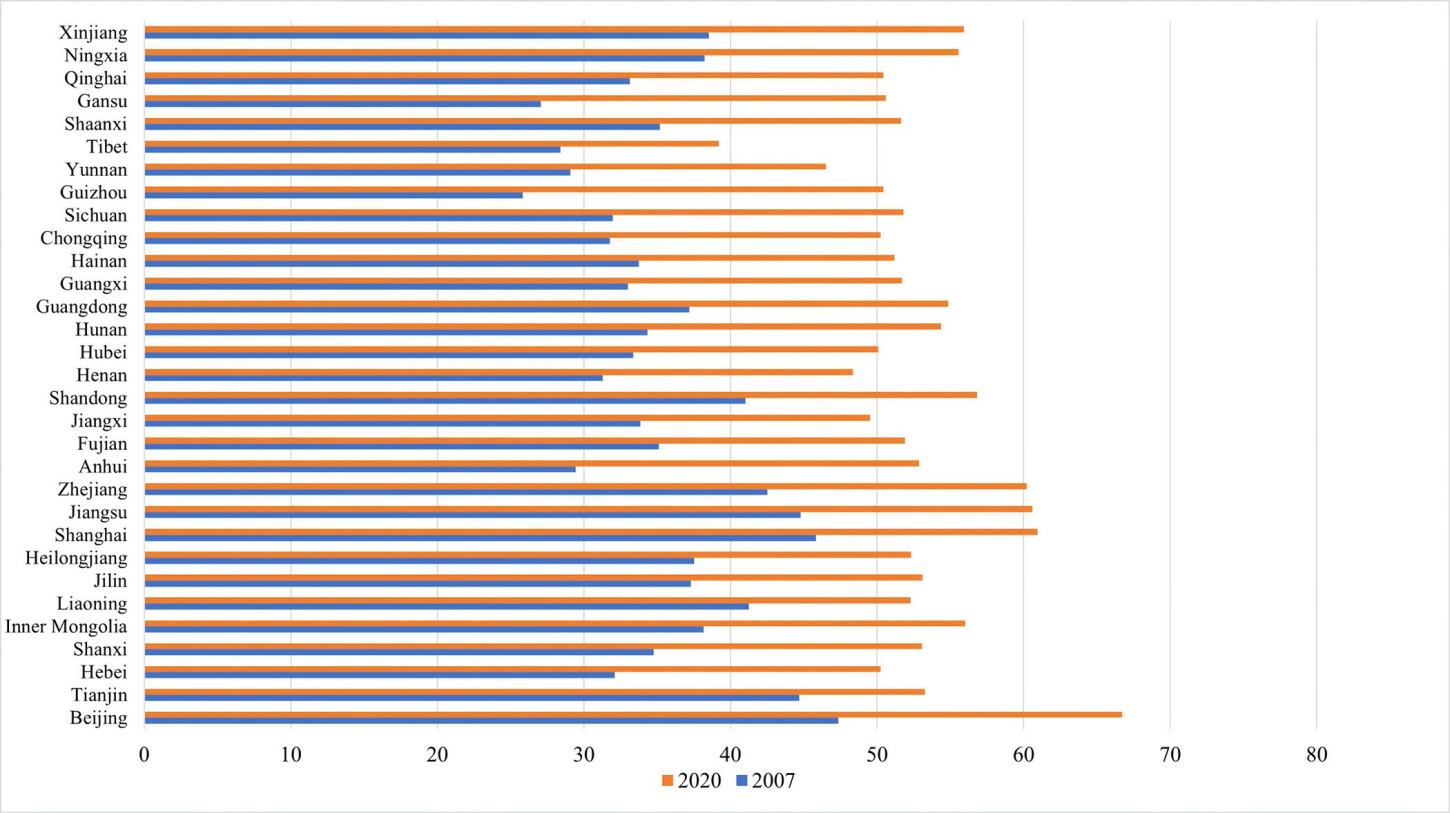
Spatial and temporal comparisons of inter-provincial levels of well-being in China.

Second, in order to clearly portray the levels of well-being in different provinces in China, this paper conducts a comparative analysis of well-being levels in the sample provinces in 2007 and 2020 (as shown in Fig 2). In terms of the overall trend of change, the level of well-being in all provinces in 2020 was significantly higher than that in 2007. In terms of the distribution pattern, the level of well-being in all provinces in 2007 was generally characterized by a “high in the east and low in the west” distribution. The provinces with the highest levels of well-being were represented by Beijing, Shanghai, Jiangsu, Tianjin and Zhejiang, and were concentrated in the developed regions of eastern China. The provinces with the lowest levels of well-being were mainly in the western region, including Yunnan, the Tibet Autonomous Region and Gansu Province. Comparing the distributional characteristics of the well-being levels of provinces in 2020 with those in 2007, it can be found that the well-being levels in the western region have increased more significantly, with Xinjiang, Ningxia, Gansu and other provinces seeing even higher increases than eastern provinces such as Liaoning and Tianjin provinces. However, at the same time, the provinces with the lowest levels of well-being are still mainly located in the western and central regions of China, including Tibet Autonomous Region, Yunnan, Henan and Jiangxi provinces. The above results show that the development of well-being levels in China’s three major regions exhibits certain convergent characteristics, but the uneven development between regions is still relatively obvious.

**Fig 3:**
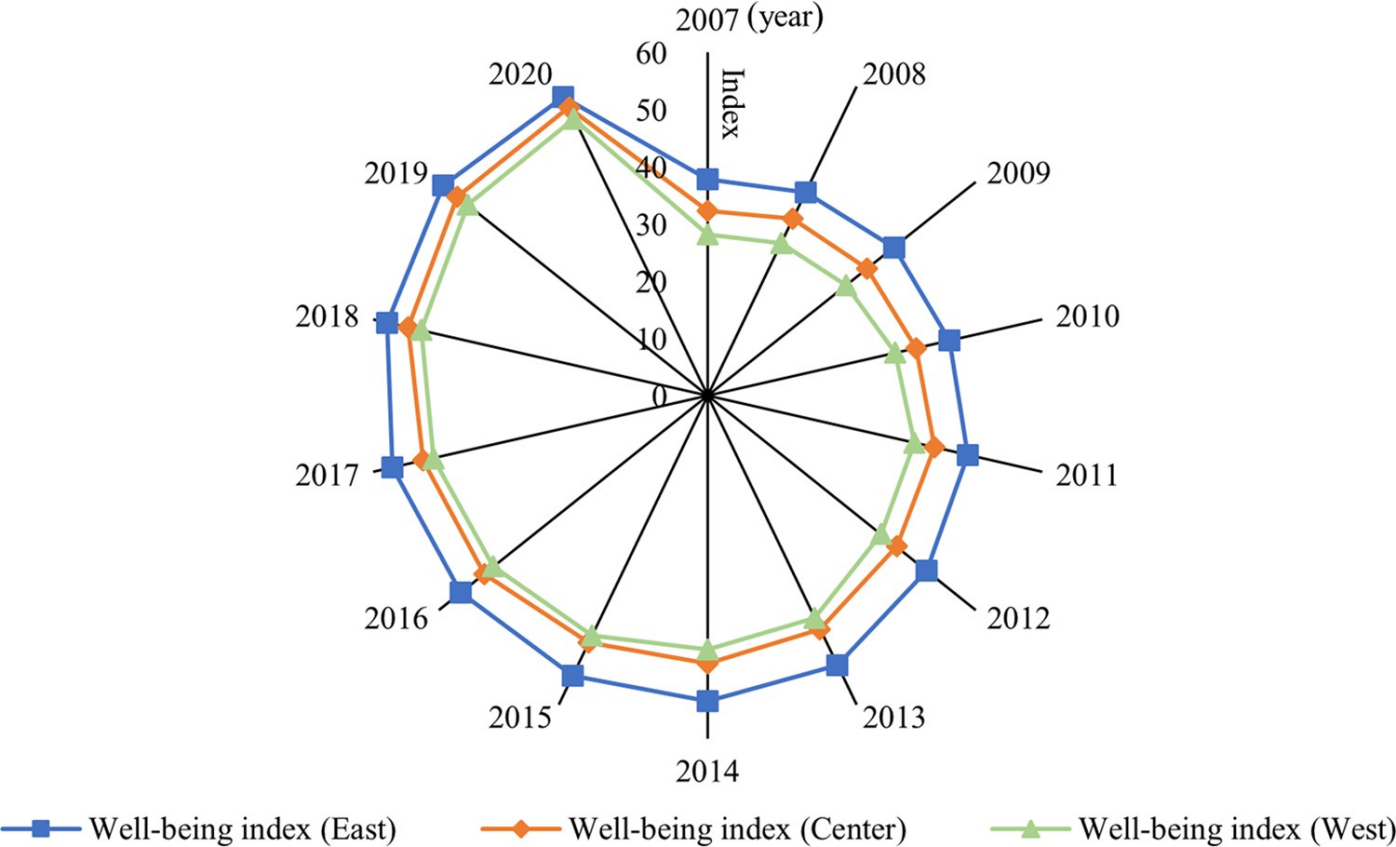
Evolution trend of the mean value of the well-being index in the three regions from 2007 to 2020.

Finally, in order to visually compare the development of well-being levels in different regions, we plot the evolution trend of the mean value of the well-being index in three regions from 2007 to 2020 (as shown in Fig 3). During the sample survey period, the trend of the well-being index in the three regions is consistent with that in the entire country, showing a significant improvement trend. From a year-by-year perspective, the well-being level in eastern China has always been in the leading position, followed by the central and western regions. In terms of the mean value, the well-being indexes of eastern, central and western regions were 50.17, 44.14 and 42.93, respectively, indicating that the well-being level showed a distribution pattern of “decreasing from east to west.” In terms of the growth rate, the average annual growth rates of the well-being index in the eastern, central and western regions were 2.56%, 3.28% and 3.50%, respectively, indicating that the central and western regions were in a period of “latecomer catching up” and that the gap between the three regions is narrowing.

**Fig 4:**
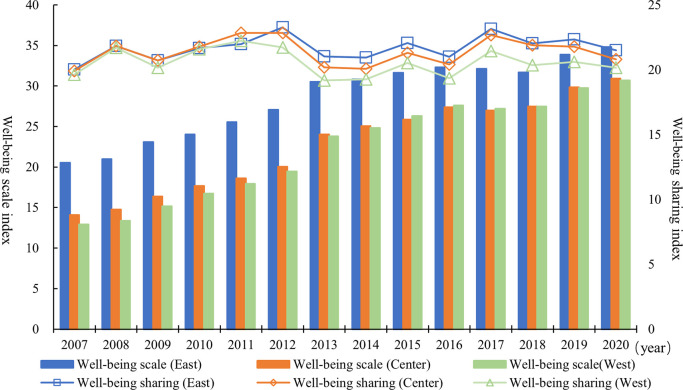
Evolution trend of the mean value of well-being’s sub-index in the three regions from 2007 to 2020.

The evolution trend of the mean value of well-being’s sub-index in the three regions from 2007 to 2020 is shown in Fig 4. From the perspective of the well-being scale index, the comparison of the annual scores, mean values and growth rates of the three regions is consistent with the regional results of the well-being level. Based on the evolutionary trend of the well-being sharing index in the three regions from a general trend perspective, the level of well-being sharing has increased in all three regions, but the magnitude of the increase has been relatively limited in each case. From a year-by-year perspective, the eastern region has led the nation in terms of well-being in most years, the central region has been largely in the middle and the western region has always been at the lowest level. In terms of mean value, the well-being sharing index of the eastern, central and western regions were 21.68, 21.36 and 20.56, respectively, indicating that the well-being sharing level also has the characteristics of “decreasing from east to west.” In terms of growth rate, the average annual growth rates of the well-being sharing index in the eastern, central and western regions were 0.55%, 0.34% and 0.20%, respectively. It indicates that the gap between the well-being sharing index of the central and western regions and that of the eastern region may widen further. The sharing level of well-being development in the three regions does not show a convergence trend.

## 4 Spatial differences of well-being level

This paper uses the method of the Dagum Gini index and its decomposition. We reveal the size and the component of spatial differences in the development of well-being in China by measuring the overall difference, intra-regional difference, inter-regional difference and the contribution rate of well-being level.

### 4.1 Overall and intra-regional difference analysis

**Fig 5:**
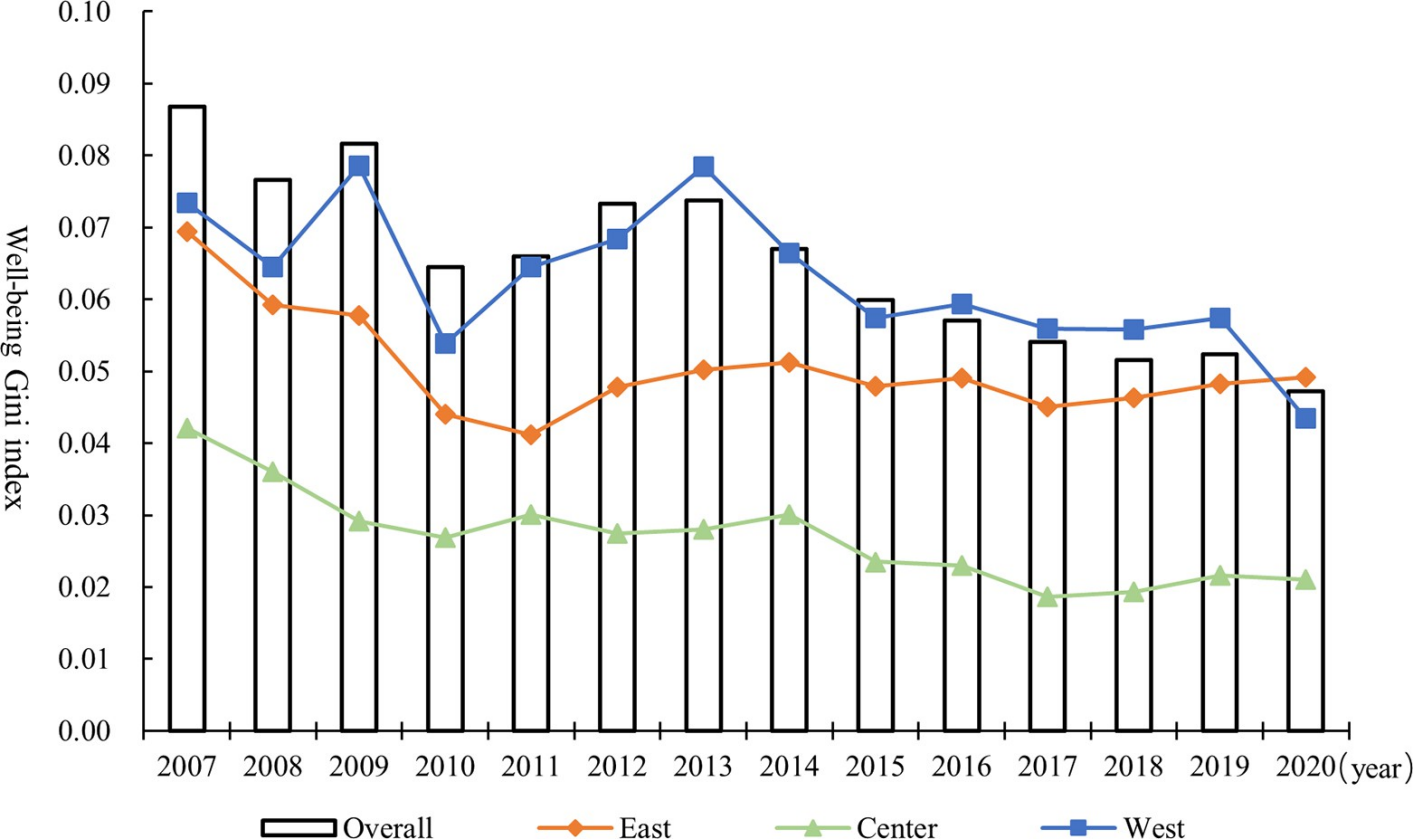
Evolution of the Gini index for the overall and three regions’ well-being levels from 2007 to 2020.

Fig 5 and S1 Table in S1 File show the evolution trend of the Gini index for China’s overall and three regions’ well-being levels from 2007 to 2020. In terms of general trends, the Gini index of the well-being levels all trended downward, which indicates that spatial imbalances have all eased. From 2007 to 2020, the overall Gini index decreased from 0.087 to 0.047, with an average annual decrease of 4.57%. This indicates that the trend of coordination at the level of well-being is increasing. By stages, the spatial differences in the levels of well-being, both overall and among the three regions, have tended to diminish amid fluctuations. Further, there are the following findings by comparing the measurement results of the three regions. First, the Gini index of well-being levels in the western region is at the highest level in most years, with the relatively highest degree of spatial imbalance, followed by the eastern and central regions, respectively. Second, the Gini index for the eastern, central and western regions have decreased by 2.62%, 5.19% and 3.95%, respectively, indicating that the spatial imbalances in the development of well-being have tended to be alleviated within the three regions.

### 4.2 Inter-regional difference analysis

**Fig 6:**
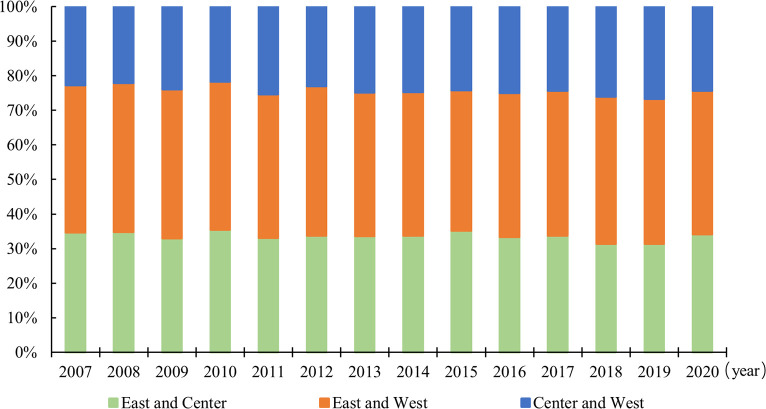
Inter-regional Gini index distribution of well-being levels in three regions from 2007 to 2020.

Fig 6 and S2 Table in S1 File report the distribution dynamics of the inter-regional Gini index for three regions’ well-being levels. First, the inter-regional Gini index of well-being levels all show a decreasing trend, indicating that the relative differences in the development of well-being have all been alleviated. Second, the differences in the level of well-being are the largest between eastern and western regions, followed by the differences between eastern-central and central-western regions, respectively. Finally, the inter-regional differences in the development of well-being in the eastern and western regions narrowed most significantly, with an average annual decrease of 5.10%. This is followed by the inter-regional differences between eastern-central and central-western regions, with an average annual decrease of 5.03% and 4.40%, respectively.

### 4.3 Component contribution analysis of spatial variation

**Fig 7:**
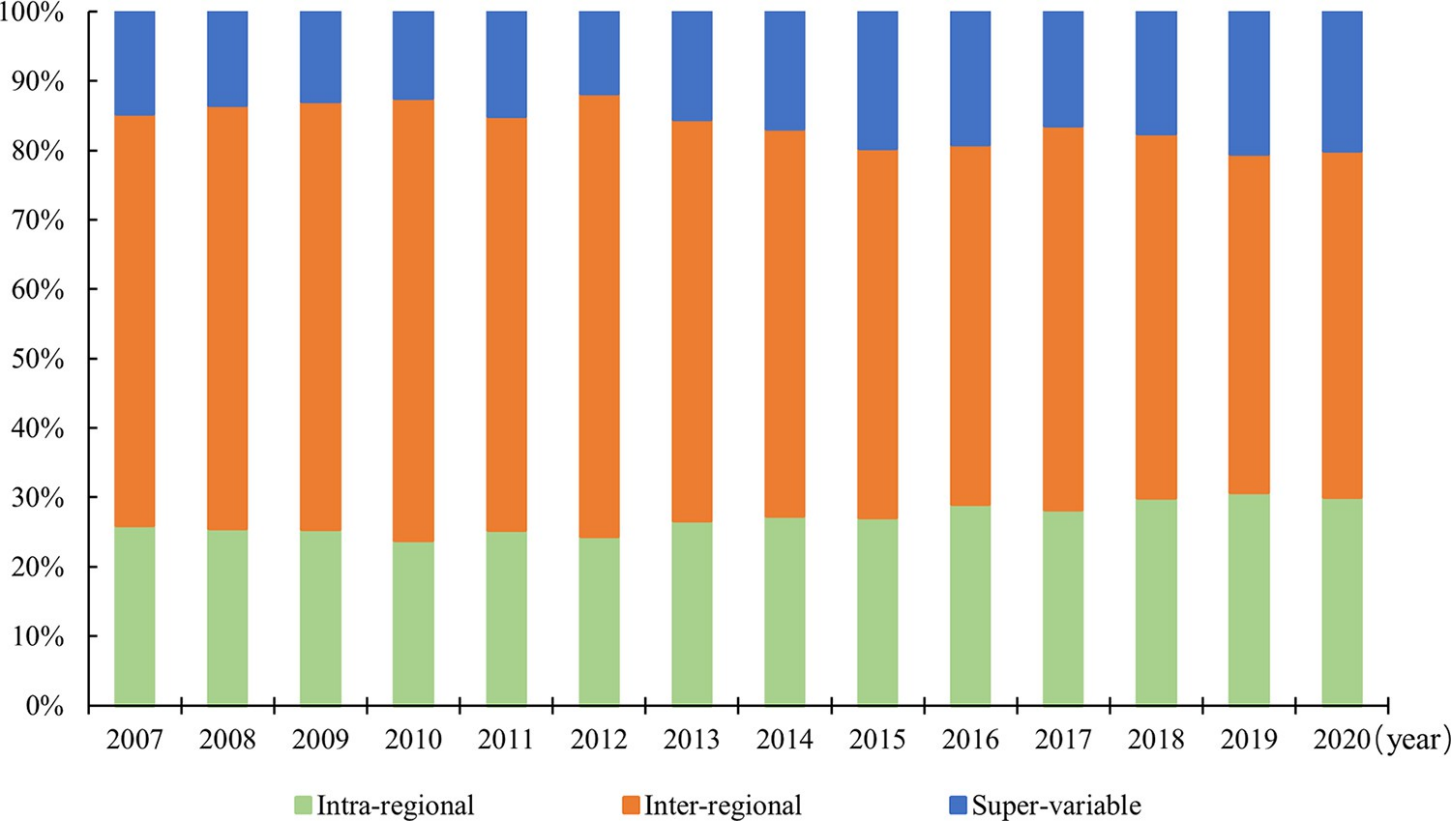
Distribution of the source contribution of spatial difference in China’s well-being level.

Table 2 and Fig 7 show the source contributions to the spatial variation in well-being and the distribution of their contribution rates in China from 2007 to 2020, respectively. First, from the contributions year by year, the inter-regional contribution is always higher than the intra-regional contribution and the super-variable density contribution, and its mean value exceeds the sum of the means of the remaining two. This indicates that inter-regional differences are the main source of spatial differences in well-being level. Second, the contribution to the overall intra-regional differences is in descending order from the western region, the eastern region and the central region. Moreover, the contributions to the intra-regional differences of the three regions all show a fluctuating downward trend. Next, the degree of contribution to the overall inter-regional differences is, in descending order, the inter-regional differences between eastern-western, between eastern-central and between central-western regions, and the contributions of all three are on a downward trend. Finally, from the dynamics of the distribution of contribution rates, the intra-regional and super-variable density contribution rates increased during the sample period, with annual average growth rates of 1.11% and 2.41%, respectively, while the inter-regional contribution rate tended to decrease, with an annual average decrease of 1.31%.

**Table 2:** Source contribution of spatial difference in China’s well-being level.

Year	Intra-regional Contribution				Inter-regional Contribution				Super-variable Density
	East	Center	West	Overall	East-Center	East-West	Center-West	Overall	
2007	0.010	0.003	0.010	0.023	0.017	0.031	0.004	0.051	0.013
2008	0.008	0.002	0.009	0.020	0.015	0.028	0.004	0.047	0.010
2009	0.008	0.002	0.011	0.021	0.016	0.030	0.005	0.050	0.011
2010	0.006	0.002	0.008	0.015	0.014	0.024	0.003	0.041	0.008
2011	0.006	0.002	0.009	0.017	0.013	0.023	0.003	0.039	0.010
2012	0.007	0.002	0.009	0.018	0.015	0.028	0.004	0.047	0.009
2013	0.007	0.002	0.011	0.020	0.015	0.025	0.003	0.043	0.011
2014	0.007	0.002	0.009	0.018	0.013	0.022	0.002	0.037	0.011
2015	0.007	0.001	0.008	0.016	0.012	0.019	0.001	0.032	0.012
2016	0.007	0.001	0.008	0.017	0.010	0.018	0.002	0.030	0.011
2017	0.006	0.001	0.008	0.015	0.010	0.018	0.002	0.030	0.009
2018	0.006	0.001	0.008	0.015	0.008	0.016	0.003	0.027	0.009
2019	0.006	0.001	0.008	0.016	0.008	0.015	0.002	0.026	0.011
2020	0.007	0.001	0.006	0.014	0.008	0.014	0.002	0.024	0.009
Mean	0.007	0.002	0.009	0.017	0.012	0.022	0.003	0.037	0.010

## 5 Structural decomposition of the well-being level’s dynamic evolution

Based on the decomposition results of Kaya identity, this paper utilizes the LMDI decomposition method to examine the drivers of dynamic changes in well-being levels from four dimensions: economic, social, ecological and technological. We analyzed the driving effects of well-being levels based on the perspective of dynamic changes, which helps identify the intrinsic driving effects of the spatial and temporal distribution of well-being levels. It can also provide a reference for improving the well-being levels of each region.

### 5.1 Structural decomposition of changes in overall well-being levels

**Fig 8:**
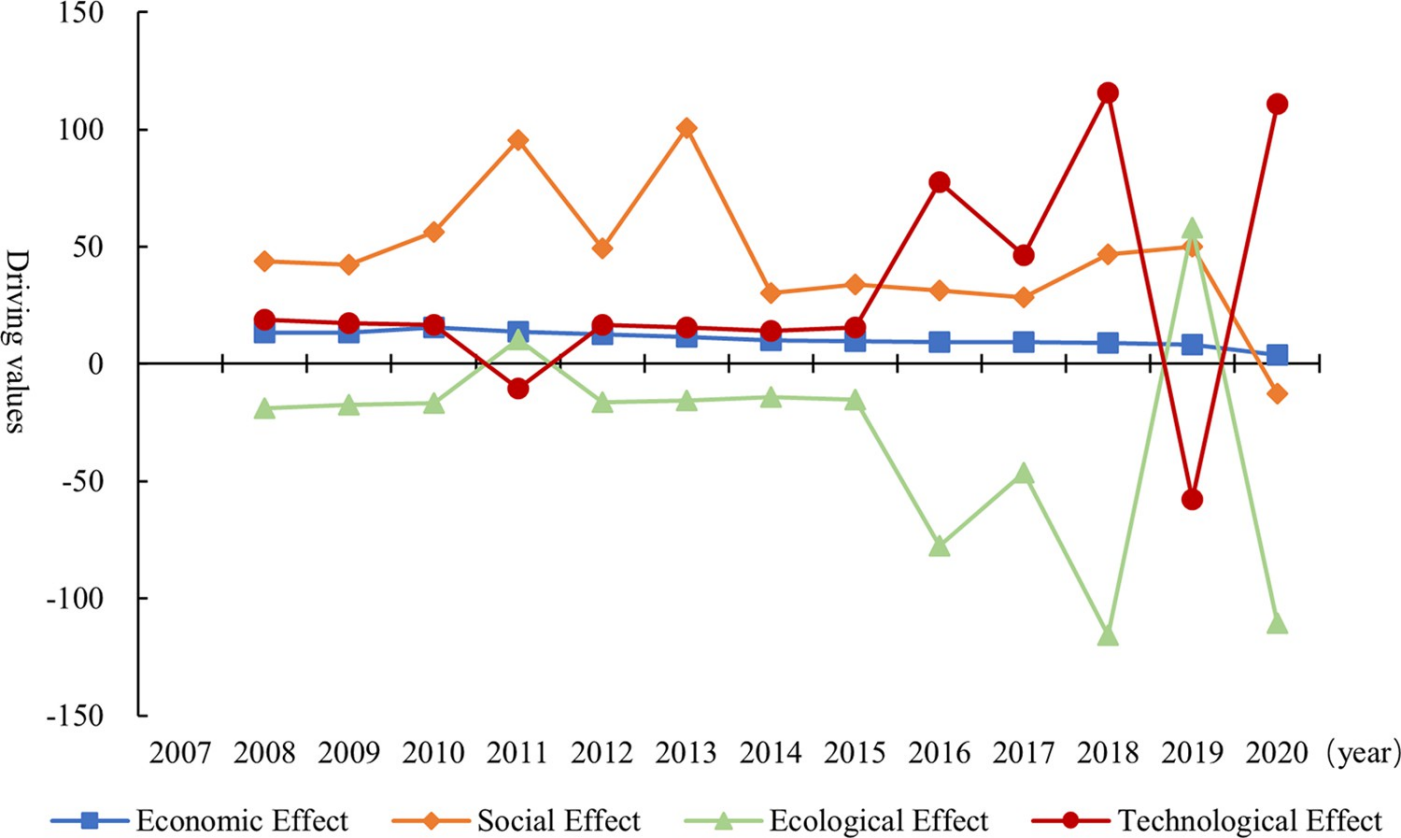
Driving effects of the dynamic evolution of well-being levels in China from 2007 to 2020.

Fig 8 and S3 Table in S1 File show the trend of driving effects evolution on the change of China’s well-being levels from 2007 to 2020 (analyzed by year spacing of 1).

#### 5.1.1 Economic effect

The economic effect on the dynamics of China’s well-being levels has always been a positive driver during the sample period. This indicates that economic growth is an important driver of sustained improvement in well-being levels. Economic construction, as a national priority, is also a necessary guarantee for solidly improving the level of well-being. China is still in the ranks of middle-income countries. As the world’s largest developing country, China still has a significant gap compared to developed countries. Consequently, the improvement of the overall well-being level requires giving full play to the key role of economic growth and the further accumulation of material wealth for a considerable period. In terms of evolutionary trends, the positive driving force of economic effects on changes in China’s well-being levels has gradually decreased since 2010. This is because after the 2008 financial crisis, China’s economy gradually shifted from a stage of high growth to a stage of high-quality development, and the central government’s policy guidance for local economic growth gradually shifted from an emphasis on economic scale to a greater emphasis on growth quality.

#### 5.1.2 Social effect

The social effect consistently had a positive driving effect on the dynamics of China’s well-being levels during 2007–2019, converging to zero only in 2019–2020. The average effect value of the social effect was 45.82 during the sample period, which was higher than the economic, ecological and technological effects. This indicates that the social effect was the main driver of the change in the level of well-being in China. From 2007 to 2020, the average annual growth rate of GDP per capita in 31 provinces averages 8.51 percent, with a subsequent increase of 3.08 percent in the average level of well-being. Hence, the growth of the GDP per unit per capita produced good social outcomes. China’s rapid urbanization is accompanied by a large influx of people into cities, which places higher demands on local governments to improve public services. This is manifested by an increase in nonproductive public goods and fiscal spending on people’s well-being, resulting in a growing synergy between improved levels of well-being and economic growth. In terms of evolutionary trends, the positive drive of the social effect from 2007 to 2019 was characterized by significant fluctuations, with an average annual increase of 1.23%, indicating that the social effect of economic growth tended to increase. However, the outbreak of COVID-19 in late 2019 led to a serious setback in economic and livelihood development. This explains why the driving effect of social effects on the level of well-being in China was markedly lower in 2020.

#### 5.1.3 Ecological effect

The ecological effect had a significant negative driving effect on the dynamics of well-being levels, with an average effect value of -30.50. This suggests that ecological stress is a driving force that hinders the improvement of well-being levels in China. This is consistent with studies that report that air pollution has a cumulative effect [50]. It will cause huge welfare losses in economic and social areas, such as consumption, investment and the physical health of the population. In recent years, Chinese government has proposed new development concepts, such as green development concepts and low-carbon concepts. However, the sloppy growth model in the early years has resulted in massive emissions of air pollutants, such as SO_2_, which has led to an increasingly severe ecological environment. Moreover, the total amount of pollutant emissions exceeds the self-cleaning capacity of the environment for a long time, leading to increased difficulty in ecological management at this stage. The “pollution first, governance later” model requires a greater cost, thus seriously offsetting the results of economic and social development. In terms of evolutionary trend, the negative driving force of ecological effect continued to decay from 2007 to 2011. The negative driving force of ecological effect increased in fluctuation from 2002 to 2018. Until 2019, China’s ecological governance efforts were quite effective, and the driving effect of the ecological effect on the level of well-being turned from negative to positive. However, the driving effect of ecological effects on well-being levels turned negative again in 2020 due to the huge impact of COVID-19 on national governance efforts.

#### 5.1.4 Technological effect

The technological effect had a significant positive driving effect on the dynamics of well-being levels in China, with an average effect value of 30.50, indicating that technological progress is an important driver of well-being enhancement. The level of well-being increased remarkably from 2007 to 2020, whereas the output per capita corresponding to the unit of environmental quality consumed in the 31 provinces increased by an average of 2.01%. Therefore, the increase in well-being levels was accompanied by higher economic gains from technological progress. The positive driving effect of technological effects on the improvement of well-being level was shown in the following aspects. On the one hand, technological advances in the ecological field help to improve the efficiency of combating environmental problems and promote the diffusion of industrial green production models, thus contributing to the improvement of environmental quality. On the other hand, science and technology innovation can promote the transformation of scientific and technological achievements, thus benefiting people’s lives by improving the intelligence level of social production and consumption.

In summary, the combination of economic, social, ecological and technological effects resulted in a gradual increase in well-being levels over the sample period. The ecological effect had a negative driving effect on the well-being level. However, the other three effects had an obvious positive driving force and were remarkably stronger than the negative driving force of the ecological effect, which provides a guarantee for the continuous improvement of well-being level. Further, among the positive drivers, social effects became the first driver to enhance the well-being level. Since 2015, after years of foreign investment, talent training and technological research and development, China’s strategy of Scientific and Technological Powerhouse has achieved remarkable results. Technological progress has become a powerful engine to drive up the level of well-being.

### 5.2 Driving effects of changes in well-being levels in the three regions

By identifying the structural factors of dynamic changes in well-being levels in the three regions, we were able to effectively clarify the differential driving characteristics of the different drivers of changes in well-being levels in each region (as shown in S4 Table in S1 File). The specific analysis is as follows (year spacing is 1).

#### 5.2.1 Economic effect

The economic effect has a positive driving effect on the change in well-being level in the three regions during the sample examination period. This indicates that economic growth is an important support for the improvement of well-being levels in China. Among them, the economic effects in the western, eastern and central regions were in decreasing order, with average effect values of 4.28, 3.63 and 2.78, respectively. This shows that the economic effect was a stronger driver of increased levels of well-being in the western and eastern regions and a weaker driver in the central region. The general trend of the change in effect values showed that the positive driving force of economic effects on the change of well-being level in all three regions tended to decrease from 2007 to 2020. Compared to the initial year, the annual average of the economic effect values in the eastern, central and western regions decreased by 3.68, 2.53 and 3.44, respectively. China’s economy is moving from a stage of rapid growth to a stage of high-quality development. The concept of development, in which regions blindly pursued economic scale, is shifting to a new stage of advancing both scale and quality.

#### 5.2.2 Social effect

The social effect also has a significant positive driving effect on the change in well-being level in the three regions during the sample examination period. This indicates that the unit economic growth in all three regions produced good social effects, which effectively drove up the well-being levels of residents. Among them, the positive social effects in the central, eastern and western regions were increasing in order, with average effect values of 11.68, 13.62 and 20.52, respectively. This suggests that social effects were a stronger driver of well-being levels in the western and eastern regions and a relatively weaker driver in the central region. The positive driving force of social effect on the change in well-being level in all three regions from 2007 to 2020 was weakening in fluctuation, and the values of social effect in the eastern, central and western regions were lower by 26.94, 15.14 and 14.59, respectively, compared with the initial years. This indicates that the driving effect of economic growth on social welfare and livelihood development has weakened in recent years. Notably, social effects were significantly stronger positive drivers of well-being levels than economic effects. This means that local governments have worked hard to promote synergistic economic and social development while meeting residents’ needs for a better life.

#### 5.2.3 Ecological effect

Ecological effects generally had a negative driving effect on changes in well-being levels in the three regions. All three regions are facing greater ecological and environmental pressure in the development process, which hinders the improvement of well-being levels. Among them, the negative drivers of ecological effects in the central, western and eastern regions were in increasing order, with average effect values of -7.24, -9.28 and -13.98, respectively. The practical reasons for this result are as follows. The eastern region is a major concentration of the Chinese population and enterprises with intensive economic activities. Whereas gaining an advantage in economic growth, it also has a stronger negative impact on resources and the environment. The western region is rich in natural resources and has a low density of population and enterprises. Because of the complex topography and weak ecological carrying capacity, the western region is less able to prevent and cope with natural disasters and environmental pollution. The central region is located between the eastern and western regions in terms of both the overall economic activity density and the ecological access threshold, so the negative driving force of its ecological effect is at the middle level. The negative driving force of ecological effects on the change of well-being levels in the three regions from 2007 to 2020 has increased in fluctuation. Notably, the driving effect of ecological effects on well-being fluctuated sharply around 2019. This suggests that ecological management efforts in the three regions have achieved some positive results in recent years, but the gains from ecological protection have been greatly offset by the negative impacts of the COVID-19 on the livelihood development of the regions. We found that measures that simply increase the intensity of environmental protection and environmental regulations are not conducive to the sustainable improvement of well-being in each region. Local governments need to adjust and optimize the environmental policy system and implementation programs according to the stage of economic development, natural and social resource endowments, and the soundness of supporting policies.

#### 5.2.4 Technological effect

The technology effect had a significant positive driving effect on the change in the level of well-being in the three regions. The economic benefits generated from the consumption of units of environmental quality in the three regions were generally high, which strongly contributed to the improvement in well-being levels. Among them, the positive driving force of technology effect in the eastern, western and central regions were in decreasing order, with average effect values of 13.98, 9.28 and 7.24, respectively. This study shows that the technology effect has a stronger positive effect on the improvement of the level of well-being in eastern region and western region, and a relatively weaker contribution to the central region. These findings are consistent with the view that the eastern region is leading in scientific research strength and the transformation rate of scientific and technological achievements, and that the western region has more room for improvement in science and technology for the people. The driving force of technological effects on changes in well-being levels has basically maintained a fluctuating upward trend from 2007 to 2018. The study shows that the level of scientific and technological development in the three major regions of China has not only made a qualitative leap, but also achieved comprehensive development in the practical areas of technological support for the improvement of residents’ livelihoods. However, in terms of the ability to transform technological achievements into real benefits, China still has huge room for improvement, which is an important reason for the upward and downward fluctuations in the driving force of technological effects.

## 6 Conclusion and policy implications

This paper constructs a system of well-being index in China based on two dimensions: well-being scale and well-being sharing. It measures the level of provincial well-being from 2007 to 2020 and analyzes its spatial and temporal evolution, spatial differences and driving effects. The main research conclusions are as follows. First, the study of spatial and temporal distribution characteristics shows that, from the perspective of the country as a whole and of the three major regions of eastern, central and western China, the level of well-being in China has continued to rise, with the level of well-being in the western region rising at a faster rate. Meanwhile, the spatial distribution of well-being levels among the provinces in China is uneven, characterized by a pattern of “higher levels in the eastern provinces and lower levels in the western provinces”. Second, the results of the calculation and decomposition of spatial differences show that spatial differences in China’s well-being levels are tending to narrow in the overall, intra-regional and inter-regional dimensions, and that the main reason for the overall differences lies in the long-standing inter-regional differences. In addition, the largest intra-regional differences in well-being levels are in the western region, while the largest inter-regional differences are between the eastern and western regions. Finally, from the perspective of the country as a whole and the three major regions of eastern, central and western China, the dynamic improvement of well-being level is mainly driven by economic, social and technological effects, while ecological effects have a negative driving influence on changes in well-being level. Among them, social factors have long been the first driver of well-being, and the role of technological factors is gradually increasing.

The findings of this paper provide empirical evidence to comprehensively grasp the spatial and temporal evolution, spatial differences and drivers of well-being in China. On the question of “how to better promote the development of well-being in China”, following policy implications are proposed.

First, China’s well-being level increased by 29.61% during 2007–2013, but only by 12.56% during 2014–2020, suggesting that the rate of well-being development has decreased in recent years. Therefore, the Chinese government can continue to improve the basic economic system and promote the healthy development of the public and non-public economies. It provides a guarantee for the continued expansion of the scale of residents’ well-being. For state-owned enterprises concerned with well-being, the government should encourage and support their technological innovations to provide residents with more diverse products and services. State-owned enterprises in the field of culture and leisure should focus on optimizing their product supply structure to enrich residents’ spiritual and cultural life. For non-publicly owned economies, such as the individual and private sectors, the government can enhance their competitive vitality by optimizing the market environment, supporting the development of new business forms and strengthening the rule of law. In this way, they are encouraged to play a more active role in social production.

Second, the paper finds that there are significant regional differences in China’s well-being level. The average contribution of inter-regional and intra-regional differences to the overall differences exceeds 56% and 26%, respectively. Therefore, the government can make narrowing the development gap an important task in promoting the level of well-being. Specific measures could be considered in the following areas: coordinated regional development, integrated development of urban-rural areas and reform of income distribution system. Local governments have improved regional cooperation mechanisms with the aim of enhancing internal development dynamics and expanding the radiation-driven role of developed regions. They have established mechanisms for linking urban-rural areas by fostering specialized industries and improving municipal facility systems to improve the well-being levels of urban-rural areas. Focusing on narrowing the income gap between groups and between urban-rural areas, government has accelerated the formation of a supporting institutional system for key issues such as urbanization, employment and reform of the land system, with a view to enhancing fairness and reasonableness in the areas of initial distribution and redistribution.

Finally, social, ecological and technological effect drivers of well-being all have significant fluctuating variations except economic effects. Ecological effects are driven negatively. Therefore, government departments should deal with the relationship between economic development, livelihood improvement and ecological protection while promoting economic growth. On the one hand, the government insists on improving residents’ livelihoods during development and consolidating the fundamental role of economic growth in enhancing well-being. Meanwhile, the government has taken the equalization of public services as its focus, so that the improvement of well-being can keep pace with economic growth. On the other hand, the government has adopted the principle of green development to reduce the negative impacts of economic growth on the ecological environment by formulating economic development policies and implementing ecological protection measures in accordance with local conditions. In addition, the government and enterprises should enhance the positive role of science and technology in benefiting the lives of residents and expand the coverage of the fruits of development. This will enable residents to obtain more convenience and satisfaction with modern technology.

## 7 Discussion

This study constructs an indicator system that can reflect China’s economic and social development and government planning objectives. It also quantitatively analyzes the level of well-being in China since 2007. The paper not only characterizes in detail the spatial and temporal distribution of well-being, but also examines its regional differences and driving effects. Compared with other studies, the main contributions of this article are reflected in the following aspects. First, rather than placing the research perspective on pure economic gains such as GDP and productivity, this paper takes residents’ well-being and livelihood as its core content, which can provide some empirical evidence for the international community to jointly promote better development of human society. Second, the system of well-being index in this paper not only includes the economies of scale brought about by development, but also considers the actual extent to which the fruits of development are shared by the country’s residents. This provides new ideas for scientifically constructing evaluation indicators of the comprehensive strength of developing countries. Third, this paper expands the factor decomposition method commonly used in the study of environmental issues and applies it to the study of well-being drivers. Compared with causality studies that examine the influence effects of external factors, this study may be a useful attempt to explore the pathways of well-being enhancement from the perspective of internal drivers.

It needs to be recognized that this paper still has some work to be improved in future research. Due to some difficulties in acquiring data, this paper has only calculated the well-being index from a provincial perspective. The results obtained have limited precision in reflecting the regional distribution characteristics of livelihood development. In future research, we will strive to obtain more micro and refined data. As the level of economic and social development increases and residents’ preferences diversify, the index system and calculation method of well-being can be appropriately adjusted and improved, which is also an important direction for our future research.

## Supporting information

S1 File (DOCX)

S1 Data (XLSX)
