# Supplementary material for: Measurement, spatial differences and driving effects of well-being levels in China
Source: PLoS One. 2024 Oct 1;19(10):e0311291. doi: 10.1371/journal.pone.0311291 (PMC11444413; doi:10.1371/journal.pone.0311291)
Supplement: S1 File — (DOCX) [file pone.0311291.s001.docx]

**Supporting information**

**S1 Table. Gini index of well-being levels in the China’s overall and three regions**

| Year | Overall | Eastern Region | Central Region | Western Region |
| --- | --- | --- | --- | --- |
| 2007 | 0.087 | 0.069 | 0.042 | 0.073 |
| 2008 | 0.077 | 0.059 | 0.036 | 0.065 |
| 2009 | 0.082 | 0.058 | 0.029 | 0.079 |
| 2010 | 0.064 | 0.044 | 0.027 | 0.054 |
| 2011 | 0.066 | 0.041 | 0.030 | 0.065 |
| 2012 | 0.073 | 0.048 | 0.027 | 0.068 |
| 2013 | 0.074 | 0.050 | 0.028 | 0.078 |
| 2014 | 0.067 | 0.051 | 0.030 | 0.066 |
| 2015 | 0.060 | 0.048 | 0.023 | 0.057 |
| 2016 | 0.057 | 0.049 | 0.023 | 0.059 |
| 2017 | 0.054 | 0.045 | 0.019 | 0.056 |
| 2018 | 0.052 | 0.046 | 0.019 | 0.056 |
| 2019 | 0.052 | 0.048 | 0.022 | 0.057 |
| 2020 | 0.047 | 0.049 | 0.021 | 0.043 |
| Mean | 0.065 | 0.050 | 0.027 | 0.063 |

**S2 Table. Inter-regional Gini Index of well-being levels in China’s three regions**

| Year | Eastern and Central Regions | Eastern and Western Regions | Central and Western Regions |
| --- | --- | --- | --- |
| 2007 | 0.098 | 0.120 | 0.064 |
| 2008 | 0.087 | 0.108 | 0.055 |
| 2009 | 0.088 | 0.115 | 0.064 |
| 2010 | 0.076 | 0.092 | 0.047 |
| 2011 | 0.072 | 0.090 | 0.055 |
| 2012 | 0.082 | 0.105 | 0.056 |
| 2013 | 0.080 | 0.099 | 0.059 |
| 2014 | 0.073 | 0.089 | 0.053 |
| 2015 | 0.068 | 0.079 | 0.047 |
| 2016 | 0.060 | 0.074 | 0.045 |
| 2017 | 0.058 | 0.072 | 0.041 |
| 2018 | 0.050 | 0.068 | 0.041 |
| 2019 | 0.050 | 0.067 | 0.043 |
| 2020 | 0.050 | 0.061 | 0.036 |
| Mean | 0.071 | 0.089 | 0.050 |

**S3 Table. Driving effects of changes in well-being levels (*∆WI*) from 2007 to 2020**

| Time Period | Economic effect | Social effect | Ecological effect | Technological effect | *∆WI* |
| --- | --- | --- | --- | --- | --- |
| 2007~2008 | 13.348 | 43.854 | -18.981 | 18.981 | 57.202 |
| 2008~2009 | 13.322 | 42.290 | -17.358 | 17.358 | 55.612 |
| 2009~2010 | 15.397 | 56.219 | -16.657 | 16.657 | 71.615 |
| 2010~2011 | 13.598 | 95.390 | 10.411 | -10.411 | 108.988 |
| 2011~2012 | 12.599 | 49.437 | -16.458 | 16.458 | 62.037 |
| 2012~2013 | 11.648 | 100.581 | -15.589 | 15.589 | 112.229 |
| 2013~2014 | 10.093 | 30.267 | -13.981 | 13.981 | 40.360 |
| 2014~2015 | 9.720 | 33.837 | -15.388 | 15.388 | 43.558 |
| 2015~2016 | 9.361 | 31.459 | -77.568 | 77.568 | 40.820 |
| 2016~2017 | 9.183 | 28.251 | -46.382 | 46.382 | 37.434 |
| 2017~2018 | 8.795 | 46.752 | -115.776 | 115.776 | 55.547 |
| 2018~2019 | 8.238 | 50.153 | 57.943 | -57.943 | 58.391 |
| 2019~2020 | 3.691 | -12.817 | -110.704 | 110.704 | -9.127 |

**S4 Table. Driving effects of changes in well-being levels (*∆WI*) in China’s three regions from 2007 to 2020**

| Region | Time Period | Economic effect | Social effect | Ecological effect | Technological effect | *∆WI* |
| --- | --- | --- | --- | --- | --- | --- |
| Eastern Region | 2007~2008 | 4.796 | 14.269 | -9.420 | 9.420 | 19.065 |
|  | 2008~2009 | 4.843 | 20.190 | -7.683 | 7.683 | 25.032 |
|  | 2009~2010 | 5.621 | 14.463 | -4.904 | 4.904 | 20.083 |
|  | 2010~2011 | 4.086 | 32.452 | 2.091 | -2.091 | 36.538 |
|  | 2011~2012 | 3.905 | 22.944 | -5.575 | 5.575 | 26.849 |
|  | 2012~2013 | 3.943 | 32.437 | -6.108 | 6.108 | 36.380 |
|  | 2013~2014 | 3.461 | 8.684 | -5.706 | 5.706 | 12.145 |
|  | 2014~2015 | 3.371 | 6.273 | -6.475 | 6.475 | 9.643 |
|  | 2015~2016 | 3.049 | 5.252 | -33.231 | 33.231 | 8.301 |
|  | 2016~2017 | 3.125 | 12.447 | -25.021 | 25.021 | 15.572 |
|  | 2017~2018 | 3.041 | 5.671 | -55.436 | 55.436 | 8.712 |
|  | 2018~2019 | 2.873 | 14.669 | 25.267 | -25.267 | 17.542 |
|  | 2019~2020 | 1.114 | -12.667 | -49.500 | 49.500 | -11.553 |
| Central Region | 2007~2008 | 3.473 | 14.213 | -4.753 | 4.753 | 17.686 |
|  | 2008~2009 | 3.340 | 7.723 | -4.450 | 4.450 | 11.063 |
|  | 2009~2010 | 3.986 | 10.549 | -3.971 | 3.971 | 14.535 |
|  | 2010~2011 | 3.699 | 22.746 | 1.089 | -1.089 | 26.445 |
|  | 2011~2012 | 3.361 | 9.719 | -4.449 | 4.449 | 13.080 |
|  | 2012~2013 | 2.917 | 21.153 | -3.779 | 3.779 | 24.071 |
|  | 2013~2014 | 2.503 | 9.405 | -3.352 | 3.352 | 11.908 |
|  | 2014~2015 | 2.344 | 5.566 | -3.457 | 3.457 | 7.910 |
|  | 2015~2016 | 2.452 | 14.485 | -19.998 | 19.998 | 16.937 |
|  | 2016~2017 | 2.530 | 5.508 | -10.138 | 10.138 | 8.038 |
|  | 2017~2018 | 2.382 | 17.952 | -24.348 | 24.348 | 20.334 |
|  | 2018~2019 | 2.224 | 13.733 | 15.857 | -15.857 | 15.957 |
|  | 2019~2020 | 0.939 | -0.929 | -28.379 | 28.379 | 0.010 |
| Western Region | 2007~2008 | 5.079 | 15.372 | -4.808 | 4.808 | 20.451 |
|  | 2008~2009 | 5.139 | 14.377 | -5.225 | 5.225 | 19.516 |
|  | 2009~2010 | 5.790 | 31.207 | -7.783 | 7.783 | 36.997 |
|  | 2010~2011 | 5.813 | 40.191 | 7.231 | -7.231 | 46.005 |
|  | 2011~2012 | 5.334 | 16.774 | -6.434 | 6.434 | 22.107 |
|  | 2012~2013 | 4.787 | 46.991 | -5.702 | 5.702 | 51.778 |
|  | 2013~2014 | 4.129 | 12.178 | -4.923 | 4.923 | 16.306 |
|  | 2014~2015 | 4.006 | 21.998 | -5.457 | 5.457 | 26.004 |
|  | 2015~2016 | 3.860 | 11.722 | -24.340 | 24.340 | 15.582 |
|  | 2016~2017 | 3.528 | 10.296 | -11.222 | 11.222 | 13.825 |
|  | 2017~2018 | 3.372 | 23.129 | -35.993 | 35.993 | 26.501 |
|  | 2018~2019 | 3.141 | 21.751 | 16.819 | -16.819 | 24.892 |
|  | 2019~2020 | 1.637 | 0.778 | -32.825 | 32.825 | 2.416 |
